# A Case of Disseminated Herpes Zoster Presenting as Vesicles Limited to Skin Lesions with Lymphoma Cutis Involvement

**DOI:** 10.3390/children8110976

**Published:** 2021-10-28

**Authors:** Joo Yup Lee, Hyun Mi Kang, Seong Koo Kim, Jae Wook Lee, Nack-Gyun Chung, Dae Chul Jeong, Bin Cho

**Affiliations:** Department of Pediatrics, College of Medicine, The Catholic University of Korea, Seoul 06591, Korea; yupsama@naver.com (J.Y.L.); ksk3497@catholic.ac.kr (S.K.K.); dashwood@catholic.ac.kr (J.W.L.); cngped@catholic.ac.kr (N.-G.C.); dcjeong@catholic.ac.kr (D.C.J.); chobinkr@catholic.ac.kr (B.C.)

**Keywords:** immunocompromised, varicella zoster virus, reactivation

## Abstract

After primary infection, varicella zoster virus (VZV) causes prolonged latent infections that may reactivate, depending on the immunologic status of the host. We present a case of VZV reactivation in a 10-year-old male patient that underwent unrelated peripheral blood stem cell transplantation (uPBSCT) for T-lymphoblastic lymphoma with lymphoma cutis lesions. This patient had a history of herpes zoster involving the right L2-5 dermatome and trigeminal V1 dermatome prior to uPBSCT. Three months post-uPBSCT, the patient’s underlying disease relapsed, and the patient presented with lymphoma cutis lesions. A few days after a skin biopsy was performed to pathologically confirm skin relapse, vesicles appeared only involving the skin areas with lymphoma cutis. This case illustrates how decreased areas of epidermal immune mechanisms may cause atypical presentations of varicella infection.

## 1. Introduction

Varicella zoster virus (VZV) is a member of the family Herpesviridae, along with herpes simplex virus, Epstein-Barr virus, and cytomegalovirus. The most significant characteristic of the Herpesviridae family is that after primary infection, they cause prolonged latent infections that may reactivate, depending on the immunologic status of the host [1].

The seroprevalence rates of varicella zoster virus (VZV) is >90% in most populations. For this reason, acyclovir is used as a prophylactic agent to suppress the reactivation of VZV after allo-hematopoietic stem cell transplantation (HSCT) for up to 1 year in adults and adolescents [2]. However, upon reactivation, the spectrum which they manifest range from asymptomatic shedding to severe and fatal diseases. Thus, during the prolonged and severe suppression of both cellular and humoral immunity in patients undergoing allo-HSCT, the reactivation of VZV may have detrimental outcomes [3]. Understanding the pathogenesis of VZV reactivation can lead to early recognition and timely intervention, which are crucial in reducing morbidity and mortality.

We present an atypical case of disseminated herpes zoster in a 10-year-old male patient that underwent unrelated peripheral blood stem cell transplantation (uPBSCT) for T-lymphoblastic lymphoma, where VZV reactivation was only observed on skin areas with lymphoma cutis.

## 2. Case

A 10-year-old male patient was diagnosed with advanced stage T-lymphoblastic lymphoma, with extra-nodal and solid organ involvement including huge mediastinal mass and cervical/supraclavicular lymph node. The patient was treated with modified non-Hodgkin lymphoma (NHL)-Berlin–Frankfurt–Münster (BFM) protocol, which included vincristine, daunorubicine, L-asparaginase, and dexamethasone 10 mg/m^2^ instead of prednisolone 60 mg/m^2^ due to the institution’s policy. The patient also received intrathecal methotrexate/hydrocortisone/cytarabine and intrathecal methotrexate in the induction regimen. An FDG-PET follow up after induction chemotherapy showed complete metabolic response.

**Figure 1:**
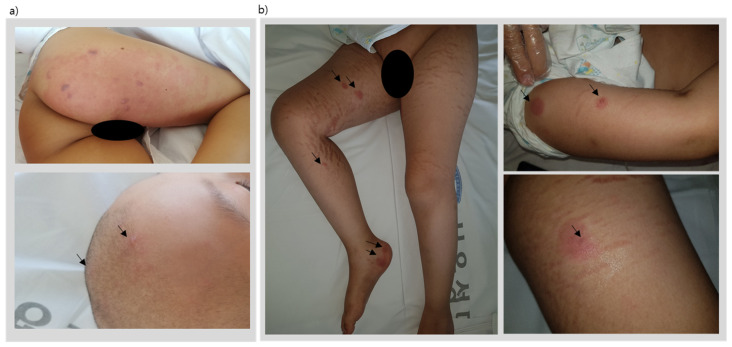
Disseminated herpes zoster lesions, (**a**) showing painful multiple erythematous vesicles on the skin involving the right L2-5 dermatome and trigeminal V1 dermatome evolving to form pustules and crusts within a course of two weeks, and (**b**) vesicles appearing only on skin areas with lymphoma cutis involvement.

After induction chemotherapy and 19 weeks of consolidation chemotherapy, the patient underwent his scheduled week-20 reinduction chemotherapy (same regimen as initial induction chemotherapy). However, on the final day of the reinduction chemotherapy protocol, the patient presented with painful multiple erythematous vesicles on the skin involving the right L2-5 dermatome and trigeminal V1 dermatome (Figure 1a) evolving to form pustules and crusts within a course of two weeks.

A vesicle fluid specimen tested positive for varicella virus via PCR; therefore, he was diagnosed with disseminated herpes zoster and treated with parenteral acyclovir. Four weeks later, a complete healing of the skin lesions was observed (Table 1).

**Table 1:** Clinical presentation of varicella zoster lesions and lymphoma cutis lesions.

Time after Diagnosis	Presentation of Skin Lesion	Symptoms	Vesicle Fluid VZV PCR	VZV IgM	VZV IgG	Skin Biopsy
6 mo	Figure 2a	Neuropathic pain involving the L2-5 dermatome skin	positive	negative	positive	-
9 mo	Figure 2b	no pain	negative	-	-	T-cell lymphoblastic lymphoma involvement
11 mo	Figure 2b	no pain	-	-	-	T-cell lymphoblastic lymphoma involvement
15 mo	uPBSCT					
3 mo post-tpl	Figure 2b	no pain	-	-	-	T-cell lymphoblastic lymphoma involvement
3 mo + 3 w Post-tpl	Figure 2c	Itching sense on the lesions	positive	negative	positive	-
5 mo Post-tpl	Figure 2b	Neuropathic pain involving the L2-5 and V1 dermatome	negative	negative	positive	T-cell lymphoblastic lymphoma involvementTissue VZV nested PCR: negative

mo, months; tpl, transplantation; uPBSCT, unrelated peripheral blood stem cell transplantation; VZV, varicella zoster virus; w, weeks.

**Figure 3:**
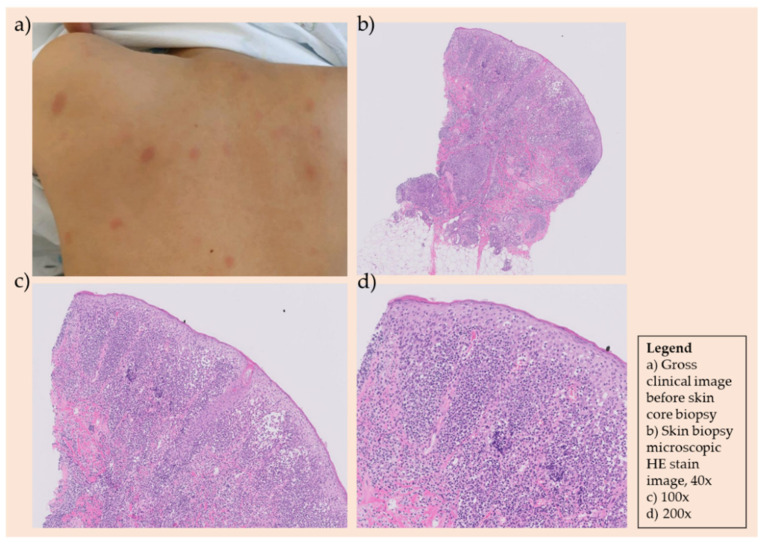
(**a**) The patient developed large macular coin-like raised skin lesions, 2–3 lesions on the scalp, several lesions on the trunk, and 2–4 lesions on each of the four extremities. (**b**–**d**) Skin biopsy showing atypical lymphoid infiltration and further immunohistochemistry stain revealing CD3+, CD5-, CD10-, CD20-, CD34-, CD99+, cyclin D1-, Ki-67 60%, TdT-, Pax-5-, with the final pathologic diagnosis as Malignant lymphoma, T-cell lymphoblastic leukemia/lymphoma at.

Nine months after initial diagnosis of T-lymphoblastic lymphoma, the patient developed large macular coin-like raised skin lesions, 2–3 lesions on the scalp, several lesions on the trunk, and 2–4 lesions on each of the four extremities. A biopsy was taken, revealing T-cell lymphoblastic lymphoma involvement of the skin (Figure 3).

The patient underwent reinduction chemotherapy with the modified NHL-BFM course AA and all the lymphoma cutis lesions disappeared. At 11 months after diagnosis, the skin lesions reappeared, and another biopsy revealed T-cell lymphoblastic lymphoma involvement of the skin (Table 1). Therefore, 15-months after initial diagnosis, the patient underwent uPBSCT for relapsed lymphoma. The donor’s VZV IgG status was unknown. The myeloablative conditioning regimen for uPBSCT included fractionated total-body irradiation (1320cGy) and cyclophosphamide. Low-dose (3.75 mg/kg) anti-thymocyte-globulin, Thymoglobuline (Sanofi-Aventis, Cambridge, MA, USA), was administered, as well as cyclosporine and methotrexate post-stem cell infusion for graft-versus-host disease prophylaxis. The lymphoma cutis skin lesions disappeared completely after uPBSCT. The patient was given 6 weeks of acyclovir prophylaxis for herpes simplex virus and varicella zoster virus (VZV) reactivation post-uPBSCT, according to the institution’s pediatric bone marrow transplant (BMT) center post-HSCT prophylaxis policy. At day 28 after uPBSCT, the patient’s post-BMT DNA test showed 99% donor chimerism, and the patient’s complete blood cell count showed total white blood cell count 7030 × 10^6^/L, absolute neutrophil count 5220 × 10^6^/L, and absolute CD3+ count 783 × 10^6^/L (absolute CD4+/CD8+ 50/514 × 10^6^/L).

Three months post-uPBSCT, the patient began showing similar skin lesions presenting as 7–10 large coin-like raised painless macules throughout the body (Table 1). Under the suspicion of lymphoma cutis relapse, another skin punch biopsy was taken. Biopsy results revealed a third recurrence of T-cell lymphoblastic lymphoma involving the skin; however, a torso FDG PET revealed markedly regressed lymphoma lesions observed at initial diagnosis. As the patient was considered refractory to treatment, a salvage chemotherapy schedule was planned. However, three weeks after a skin biopsy to pathologically confirm lymphoma cutis, the patient began to express pain only involving the lymphoma cutis skin lesions. A few days later, vesicles appeared on the lymphoma cutis lesions. Areas of the skin without any lymphoma cutis involvement were all clear of any vesicles (Figure 1b). A vesicle fluid sample was taken, and PCR results were positive for VZV. The patient was seropositive for varicella IgG at this time. Therefore, this patient was diagnosed with disseminated varicella infection and treated with parenteral acyclovir. The vesicles crusted and disappeared 7–10 days later.

However, two weeks after the disappearance of the herpetic vesicles, severe neuropathic pain refractory to parenteral analgesics occurred along the distribution of the right L2-5 dermatome and trigeminal V1 dermatome. No new vesicles were observed on the skin or lymphoma cutis lesions. However, due to the excruciating neuropathic pain that was only responsive to narcotic analgesics, the patient underwent a skin biopsy revealing negative tissue VZV nested PCR, and only evidence of T-cell lymphoblastic lymphoma involvement (tissue immunohistochemistry: ALK1-, CD3+, CD4-, CD8-, CD20-, CD30-, CD68-, CD99+, Ki-67 90%, TdT-, Pax-5-). The patient, therefore, received a nerve block of the L2-5 area, and after his neuralgia was under pain control, he was initiated with salvage chemotherapy for the treatment of relapsed T-cell lymphoblastic lymphoma.

## 3. Discussion

We present a case of an immunocompromised patient with recurrent VZV reactivation with atypical presentation. Our case highlights the importance of understanding and recognizing different clinical presentations of VZV reactivation in susceptible patients.

Many infectious complications can occur after HSCT in children with malignancies [4,5]. Varicella and herpes zoster (shingles) are two distinct clinical presentations of mucocutaneous lesions caused by VZV. After primary infection of the VZV at mucosal sites through respiratory droplets or cutaneous vesicle fluids, the virus spreads to drain the regional lymph nodes, resulting in T-cell infection and subsequent transport to other sites. Through T-cells, the virus disseminates around the body, and into peripheral blood mononuclear cells and onto epithelial cells, resulting in infection of the skin and the characteristic varicella rash. The virus then spreads to the sensory ganglia and establishes lifelong latency [1]. During the second reactivation of VZV in this patient, an atypical presentation of VZV was observed, where vesicular lesions appeared only on skin areas with lymphoma cutis. All other areas of the skin without lymphoma cutis involvement, as well as prior dermatome involvement of VZV, were clear of any vesicles or rashes.

The infiltration of malignant T-lymphocytes in the skin are known as cutaneous lymphomas, otherwise known as lymphoma cutis [6]. Through this atypical presentation, it was possible to observe that not only systemic immunity, but also local cutaneous innate immunity, is important in VZV defense. A study by Ku et al. found that increased susceptibility to memory T-cells facilitate VZV transfer to epithelial cells; however, cutaneous innate immunity was important for modulating VZV replication in the epidermal cells [7]. Furthermore, in human dermal fibroblasts, Retinoic acid-inducible gene I (RIG-1), which is a cytoplasmic viral pathogen associated with molecular patterns, senses the viruses, which results in a type I IFN response controlling VZV infection. Monocytes and other myeloid cells of the skin are also able to sense the virus [8,9]. However, decreased or altered functioning of these immune cells may result in the loss of control in VZV infections [9]. Therefore, as in this case, in lesions where the skin immunity was intact, VZV replication was not observed.

There have been case reports of lymphoma at the site of herpes zoster scars or herpes zoster mimicking lymphoma or leukemia cutis. However, to our knowledge, this is the first case report of VZV reactivation with atypical presentation of vesicles only involving lymphoma cutis lesions.

## 4. Conclusions

This case portrays the significance of recognizing atypical presentations of VZV infection in immunocompromised patients and understanding the role of cutaneous innate immunity in the modulation of VZV replication, limiting cell-to-cell transmission of the virus in epithelial cells. This case illustrates how decreased areas of epidermal immune mechanisms may cause atypical presentations of varicella infection in patients with decreased areas of cutaneous innate immunity.

## Figures and Tables

**Figure 2:**
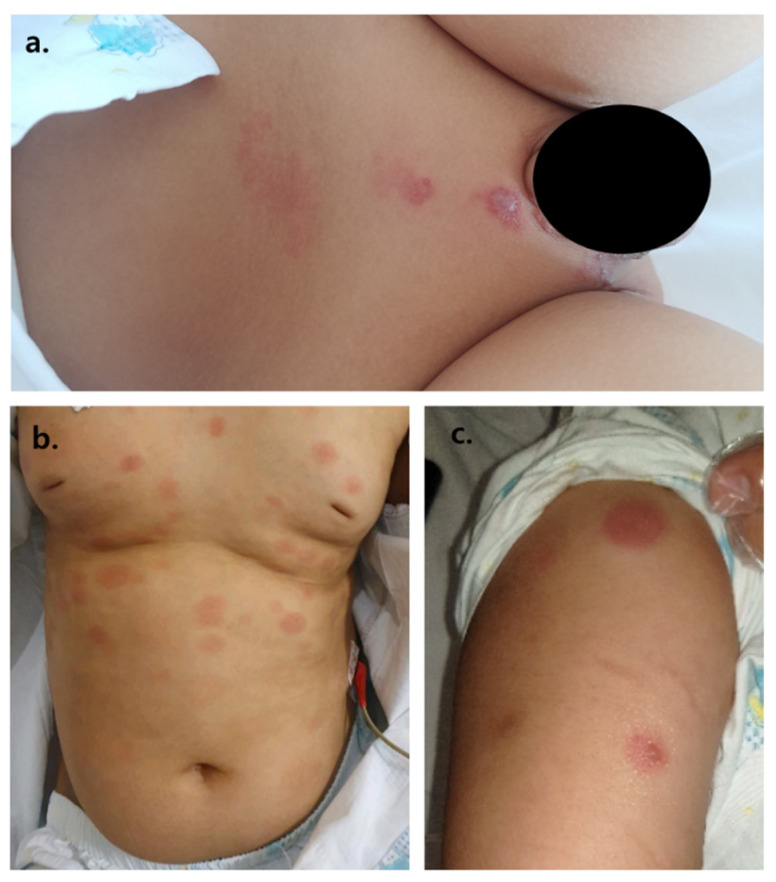
(**a**) painful erythematous rash forming vesicles, (**b**) painless coin-like, raised macules, (**c**) painful vesicles on top of painless coin-like macules.
